# Aberrant ocular architecture and function in patients with Klinefelter syndrome

**DOI:** 10.1038/s41598-017-13528-4

**Published:** 2017-10-13

**Authors:** Cristin Brand, Michael Zitzmann, Nicole Eter, Sabine Kliesch, Joachim Wistuba, Maged Alnawaiseh, Peter Heiduschka

**Affiliations:** 1 Institute of Reproductive and Regenerative Biology, Centre of Reproductive Medicine and Andrology, University of Muenster, Muenster, Germany; 2 Department of Clinical and Surgical Andrology, Centre of Reproductive Medicine and Andrology, University of Muenster, Muenster, Germany; 3 Department of Ophthalmology, University of Muenster, Muenster, Germany

## Abstract

Klinefelter Syndrome (KS), the most common chromosomal disorder in men (47,XXY), is associated with numerous comorbidities. Based on a number of isolated case reports, we performed the first systematic and comprehensive evaluation of eye health in KS patients with a focus on ocular structure and vascularization. Twenty-one KS patients and 26 male and 38 female controls underwent a variety of non-invasive examinations investigating ocular morphology (examination of retinal thickness, optic nerve head, and cornea) and function (visual field testing and quantification of ocular vessel density by optical coherence tomography angiography). In comparison to healthy controls, KS patients exhibited a smaller foveal avascular zone and a decreased retinal thickness due to a drastically thinner outer nuclear layer. The cornea of KS patients showed a decreased peripheral thickness and volume. In perimetry evaluation, KS patients required brighter stimuli and gave more irregular values. KS patients show an ocular phenotype including morphological and functional features, which is very likely caused by the supernumerary X chromosome. Thus, KS should not be limited to infertility, endocrine dysfunction, neurocognitive and psychosocial comorbidities. Defining an aberrant ocular morphology and function, awareness for possible eye problems should be raised.

## Introduction

Klinefelter Syndrome (KS) is the most common chromosome disorder in men (1: 500) that is caused by meiotic non-disjunctions in parental germ cells resulting in a supernumerary X chromosome. 80–90% of the patients exhibit a karyotype of 47,XXY, while in the remaining patients higher grade X chromosomal aneuploidies are observed^1^. KS leads to a heterogeneous phenotype and is associated with an increased morbidity and mortality^2^. Prominent features are infertility, hypogonadism, gynecomastia, disturbed bone metabolism, diabetes, neurocognitive and psychosocial comorbidities^3–6^. Hence, the predominant symptoms are of endocrine and reproductive nature, nevertheless, more comorbidities associated with aberrant vascularization have been reported recently, e.g. altered testicular vascularization, cardiovascular problems including risk for pulmonary embolism and infarction as well as altered cardiac rhythmogenic properties^7–11^. Based on these findings, we hypothesized that ocular blood flow in these patients might also be affected, since the eye is strongly perfused and reacts sensitive to vascularisation changes. Although KS is investigated since its discovery in 1942^12^, eye health and function of KS patients have never been assessed systematically beyond few isolated case reports demonstrating ocular abnormalities in 47,XXY patients^13–19^. However, whether these findings are directly related to the KS syndrome as such remains elusive. Therefore, we performed a systematic study to analyse whether KS has general ocular consequences.

For the first time, the full spectrum of non-invasive examinations was used to approach the eye health of KS patients systematically by analysing morphological (examination of retinal thickness, optic nerve head, and cornea) and functional aspects (blood flow by optical coherence tomography angiography [OCTA], and visual field testing). Taken together, our results addressed the eye health in KS patients compared to healthy controls.

## Results

### Smaller Foveal Avascular Zone in Patients with KS

**Figure 1:**
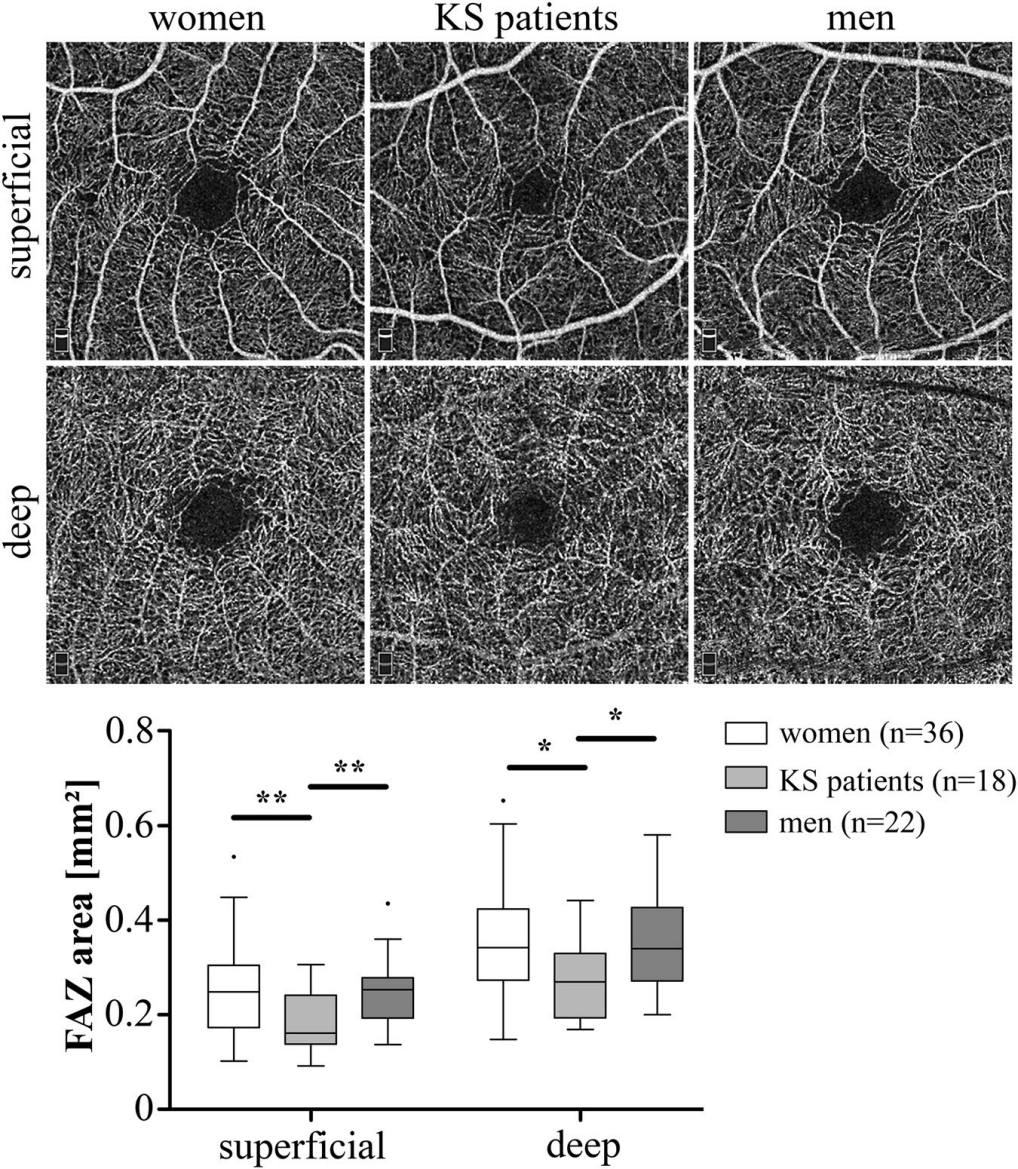
Foveal avascular zones (FAZ) in superficial and deep retinal layers of KS patients and controls. Top: Representative 3×3 mm² scans. Diagram below shows evaluation of sizes of FAZ. Levels of significance of differences between controls and KS patients are indicated by *p < 0.05 and **p < 0.01.

The size of the foveal avascular zone (FAZ) was assessed in the superficial and deep retinal layers (Fig. 1). In the superficial layer, KS patients showed a smaller FAZ (0.16 mm^2^, interquartile range [IQR]: 0.14–0.24 mm^2^) by about 35% compared to controls (women: 0.25 mm^2^, IQR: 0.17–0.30 mm^2^, p = 0.006; men: 0.25 mm^2^, IQR: 0.19–0.28 mm^2^, p = 0.009). In the deep layer, KS patients revealed also a smaller FAZ (0.27 mm^2^, IQR: 0.19–0.33 mm^2^) of approximately 21% compared to controls (women: 0.34 mm^2^, IQR: 0.27–0.42 mm^2^, p = 0.017; men: 0.34 mm^2^, IQR: 0.27–0.43 mm^2^; p = 0.017).

### Vessel Density of Patients with KS

Using OCTA, vessel density was evaluated in the angiograms of superficial and deep retinal layers and in the choriocapillaris (CC). After correction for different FAZ sizes, superficial VD values in the fovea as well as in the parafovea did not differ between groups (Table S1A). Compared to women, VD in the deep layer was lower in the majority of the areas in KS patients (p-values between 0.005 and 0.042, Table S1B). All VD values of superficial and deep layers are listed in Supplementary Table S1. VD in CC revealed no differences between KS patients and controls (KS patients 108 (IQR: 106–111), women 108 (IQR: 105–110), men 110 (IQR: 106–112)).

### Thinner Retina Due to thinner outer nuclear layer in Patients with KS

**Figure 2:**
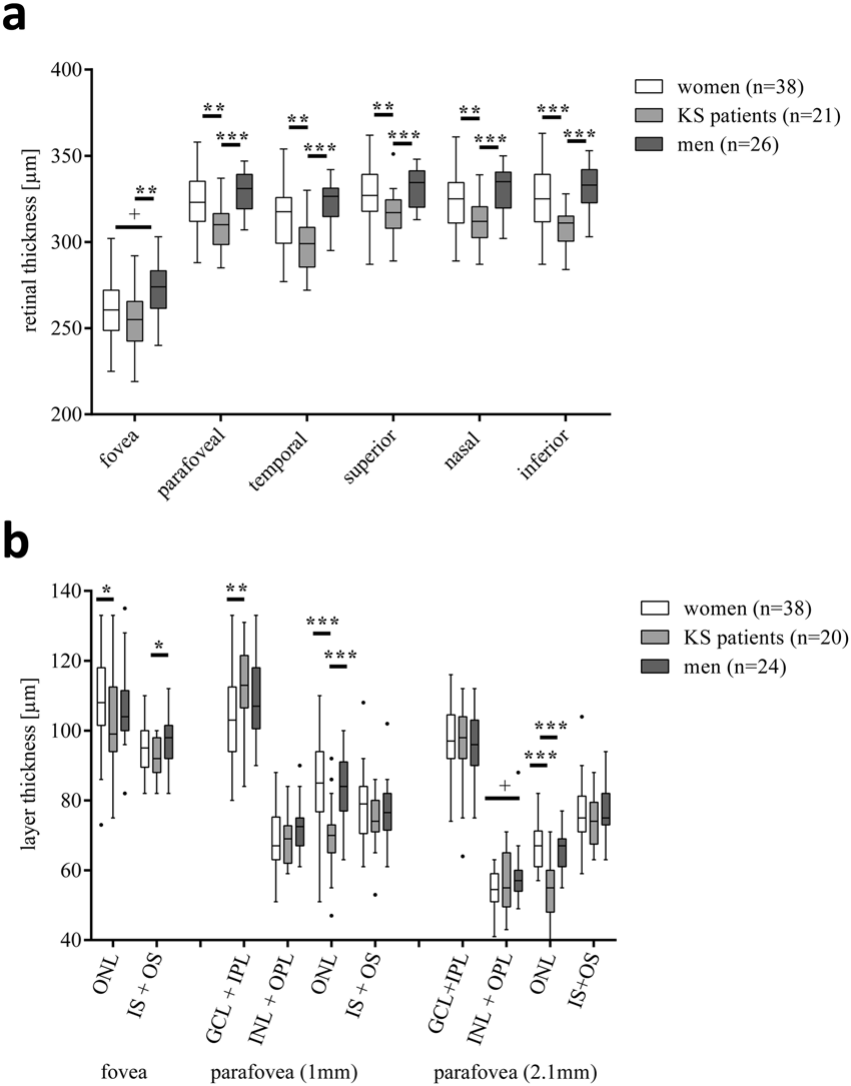
Retinal thicknesses and single layer segmentation of the fovea and the parafovea in KS patients and controls. (**a**) Retinal thickness. Parafovea was further divided into temporal, superior, nasal, and inferior areas. (**b**) Retinal segmentation (ONL: outer nuclear layer, IS + OS: photoreceptor inner, and outer segments layer, GCL: glia cell layer, IPL: inner plexiform layer, INL: inner nuclear layer, OPL: outer plexiform layer). Levels of significance of differences between controls and KS patients are indicated by *p < 0.05, **p < 0.01 and ***p < 0.001. “ + ” indicates statistically significant sex-related differences between control cohorts.

Analysis of retinal thickness revealed a substantially decreased retinal thickness by approximately 17 µm (or 5%) in all sectors in KS patients compared to female and male controls (Fig. 2A). Thickness of retinal nerve fibre layer revealed no differences between all three groups (approximately 102 µm for all three groups, data not shown). Determination of the foveal depth by assessing the ratio between foveal and parafoveal thickness showed no differences between groups (mean ratio 1.2, data not shown).

Segmentation analysis revealed a decreased thickness for the outer nuclear layer in KS patients (99 µm, IQR: 94–113 µm) compared to women (108 µm, IQR: 102–118 µm, p = 0.047, Fig. 2B). In a distance of 1 mm nasally from the fovea, the outer nuclear layer was clearly thinner in KS patients (70 µm, IQR: 65–73 µm) than in female (85 µm, IQR: 77–94 µm, p < 0.0001) and male controls (84 µm, IQR: 77–91 µm p < 0.0001). Correspondingly, segmentation analysis 2.1 mm superior-temporally from the fovea revealed also a thinner outer nuclear layer in KS patients (55 µm, IQR: 48–60 µm) compared to female (67 µm, IQR: 61–71 µm, p < 0.0001) and male controls (67 µm, IQR: 61–69 µm, p < 0.0001).

### Aberrant Corneal Parameters in Patients with KS

**Figure 3:**
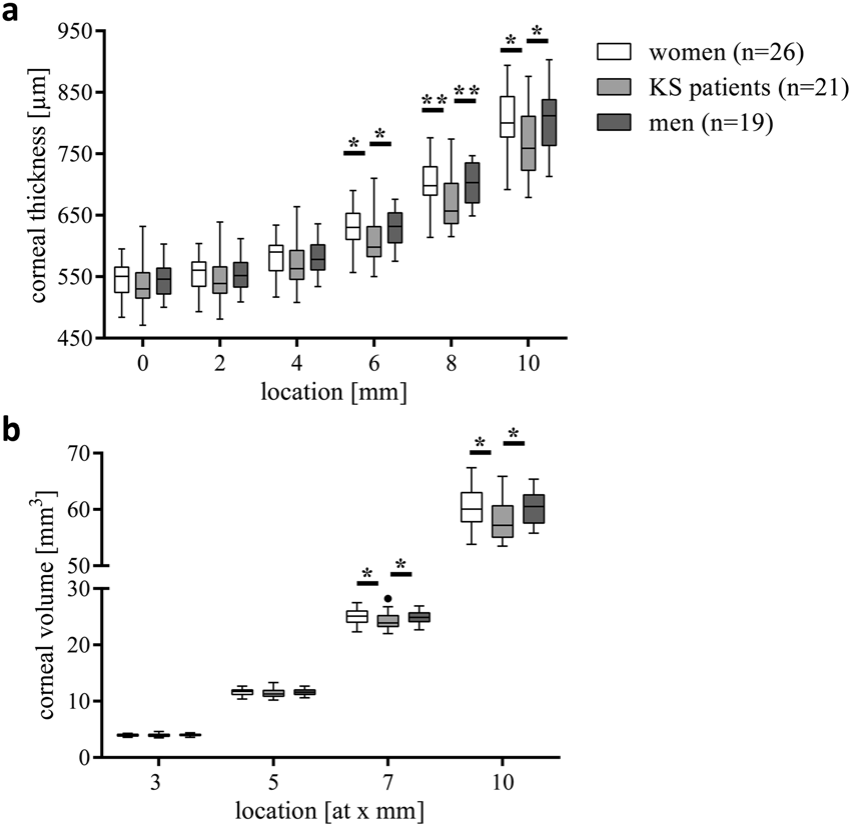
Corneal parameters in KS patients and healthy controls. Corneal thickness (CT, **a**) and corneal volume (CV, **b**). Levels of significance of differences between controls and KS patients are indicated by *p < 0.05 and **p < 0.01.

For analysis of the cornea, KS patients showed lower values for the non-central corneal thickness and volumes as depicted in Fig. 3.

### Aberrant Optic Nerve Head in Patients with KS

In the optic nerve head, VD was increased inside disc in KS patients compared to female controls (p = 0.004, Figure S1A). Analysis by HRT revealed a larger disc and rim area in KS patients (1.9 mm^2^, IQR: 1.7–2.4 mm^2^ and 1.6 mm^2^, IQR: 1.4–1.8 mm^2^) compared to women (1.8 mm^2^, IQR: 1.5–2.0 mm^2^, p = 0.030, and 1.4 mm^2^, IQR: 1.2–1.5 mm^2^, p = 0.008, Figure S1B+C and Table S2).

### Aberrant Ocular Functionality in Patients with KS

Among all studied subjects, there have been no subjects with scotoma or findings obviously “outside normal”. Visual field analysis showed a higher mean deviation (MD) and pattern standard deviation (PSD) in KS patients compared to controls (Table 1).

**Table 1:** Characteristics of the study groups (VFI: visual field index, MD: mean defect, PSD: pattern standard deviation). Levels of significance of differences between controls and KS patients are indicated by *p < 0.05 and **p < 0.001.

	Women (n = 23)	KS patients (n = 21)	Men (n = 10)
Visual acuity [logMar]	0 (0–0)	0 (0–0)	0 (0–0.1)
Spherical equivalent [D]	0 (−1.7–0.8)	0 (−1.3–0.5)	0.1 (−0.1–0.4)
VFI [%]	99.0 (98.0–99.5)	98.5 (97.0–99.5)	99.5 (98.4–99.6)
MD [dB]	−1.4 (−2.4–0.7)**	−2.5 (−3.6–1.7)	−1.3 (−2.3–0.5)*
PSD [dB]	1.6 (1.5-2.0)*	1.8 (1.7-2.4)	1.5 (1.3-1.8)**

## Discussion

We performed ophthalmological examinations in a cohort of KS patients versus healthy men and women. In general, KS patients did not notice any subjective ocular symptoms nor attract attention of ophthalmologists. Therefore, function and morphology of the eyes in KS patients have not been studied systematically so far. In this study, we found a distinct ocular phenotype associated with KS.

KS patients exhibited a smaller FAZ in the OCT angiograms of superficial and deep layers, a decreased retinal thickness, and a thinner outer nuclear layer. The anterior segment in KS patients exposed a decreased peripheral corneal thickness and volume. In perimetry, KS patients required brighter stimuli and showed a higher irregularity of values in perimetry evaluation.

After normalization for the smaller FAZ, there was no difference in relative VD between KS patients and controls in the fovea. In the parafovea, substantial differences in VD were found only between female controls and KS patients in the deep retinal layer. We also found a significant difference in the VD of the parafovea in the deep retina between female and male controls, as indicated by the symbol “+” (Table S1).

Normative VD data have been presented in different studies in the literature. Coscas *et al*. found a significantly higher VD in females when compared with males in participants older than 60 years, which could be explained by the later vascular aging in females compared to males^20^. Such differences were not found by another group^21^.

However, the eye of KS patients exhibits aberrant vascularisation, displayed by a smaller FAZ and thereby a higher absolute vascularisation in the eyes of KS patients. Blood vessels grow further into the foveal centre in KS patients and thereby enlarge the size of the vascular bed. This morphological feature may be an indicator for a dysregulation of angiogenesis during development or an aberrant requirement of the fovea in KS patients in terms of blood support. At the moment, the origin of differences in FAZ sizes is not clear so far, and the mechanism of FAZ formation is also disputed in the literature. Whereas Mintz-Hittner *et al*.^22^ published that the fovea in preterm babies was vascularised for a certain period of time, Gariano *et al*.^23^ and Provis *et al*.^24,25^ showed that the foveal region is avoided at all by growing blood vessels during development in macaques and humans.

We could show an aberrant retinal morphology that manifests in a decreased thickness and segmentation analyses revealed that this thinning was due to a reduced thickness of the outer nuclear layer, which leads to the assumption that a reduced number of photoreceptors is possibly present in the retinae of KS patients, or, less likely, the photoreceptors are more densely packed. Recent publications show anatomical deviations in another part of the central nervous system of KS patients; the brain^26^. No differences in activity of visual cortex have been described^27^. In all groups, retinal thickness increased with decreasing size of FAZ in accordance to a previously published record^28^.

Besides by CC, photoreceptors are also supplied with oxygen by the vascularisation present in the outer plexiform layer of the retina. Thus, the decreased VD we found in the OCT angiogram of the deep retinal layer compared to female controls and the thinner outer nuclear layer may lead to the speculative assumption that retinae of KS patients might contain a decreased number of photoreceptors. Thinning of the outer nuclear layer in KS patients was more pronounced in the two parafoveal regions than in the fovea. As in the fovea only cones are present and the portion of rods grows with increasing distance from the fovea, the question arises whether rods are more affected than cones by the assumed lack of photoreceptors. The increased MD and PSD values of KS patients during visual field examination might also be associated to less photoreceptors. Whether the reduction of photoreceptor number found in this study and a supposed explicit loss of rods has an effect on visual function of KS patients, in particular on the electroretinogram, remains to be elucidated in further studies.

Accurate corneal thickness measurements has become extremely important in the assessment of different diseases e.g. refractive surgery, glaucoma and keratoconus^29,30^. This study shows for the first time that KS patients reveal a decreased peripheral corneal thickness and volume compared to healthy controls. Central corneal thicknesses of controls were comparable to the literature^31,32^.

VD of ONH might reveal higher values in KS patients in comparison to female controls due to larger vessels (i.e. central retinal artery and vein) that enter and exit the eye through the ONH. Similar to the smaller FAZ, a larger disc and thus larger blind spot (+15%) detected in KS patients might also lead to a size reduction of the visual field.

Majority of KS patients received testosterone substitution by application of a transdermal testosterone gel. They were on this medication for at least 2 years. There is no theoretical background within literature how testosterone supplementation could influence the architecture of the eye in the way we describe it. The effect is, assumably, rather attributable to variation in gene dosage inherent to the 47,XXY karyotype.

Although the cohorts analysed were relatively small, results revealed a clear phenotype. Nevertheless, further investigations are needed focussing on ocular functionality and the influence of ageing or comorbidities of KS on the aberrant eye condition.

As mentioned in the Introduction, there are several cases of ocular problems in KS patients, such as choroidal atrophy^13,15^, iris coloboma^14^ or iris malformation^16^, cataract^16^, ocular hypertension^17,18^ or diabetic retinopathy^19^, just to name a few. However, it is difficult to suggest a correlation between these problems and the Klinefelter syndrome, because these problems arise also in many healthy men and women, and the link between these problems and the presence of one or more additional X chromosomes is not known. Moreover, eyes of KS patients participating in our study did not show any obvious ocular irregularities or eye diseases, and we still found the described differences to eyes of healthy controls. We do not see any correlations between a thinner cornea, a thinner retina or a smaller avascular zone to the anecdotic cases of ocular problems in KS patients mentioned above.

The presence of a supernumerary X chromosome is associated with an ocular manifestation in KS patients including a number of morphological and functional features. This should raise awareness towards a possibly higher risk of KS patients to suffer from ocular diseases with the subsequent need for different standards of care for these patients. There is need for future (epidemiological) studies on eye health of KS patients, e.g. on potential reduction of visual acuity, impaired night vision and increased risk for ocular diseases. This chromosomal condition has multifaceted consequences. Physicians, including ophthalmologists, treating KS patients should be aware that KS is a systemic disorder and patients should be examined for possible manifestations.

## Materials and Methods

### Study Design and Participants

For this prospective cohort study, 21 KS patients were recruited at the Centre of Reproductive Medicine and Andrology. Karyotypes were determined as previously described^11^. Patients presented with testosterone levels within the normal range (11.8 nmol/l, IQR 7.6–17.9 nmol/l), either spontaneously (n = 3) or by testosterone substitution (n = 18). The KS patient cohort (46 years, range = 21–60 years) was compared to 26 male controls (43 years, range = 22–70 years) and 38 female controls (46 years, range = 20–71 years) of a similar age with no history of any other systemic or ocular diseases or surgery. In addition to a male control, KS patients were also compared to healthy women to control for the second X chromosome. Although women as well as KS patients inactivate the 2^nd^ X chromosome, some genes escape this X inactivation and are still expressed^33^. All study participants were of Caucasian origin. Only one eye of each patient was randomly included into the study. Subjects with refractive error > −4 or >  + 4 D spherical equivalent or media opacities that prevented high-quality imaging were excluded. The study followed the tenets of the Declaration of Helsinki and was carried out in accordance with the relevant guidelines and regulations. Before OCTA imaging, participants gave informed consent (approval by the Ethics Committee of the University of Muenster, Germany, 2015–402-f-S and 4INie).

### Ocular Examinations

**Figure 4:**
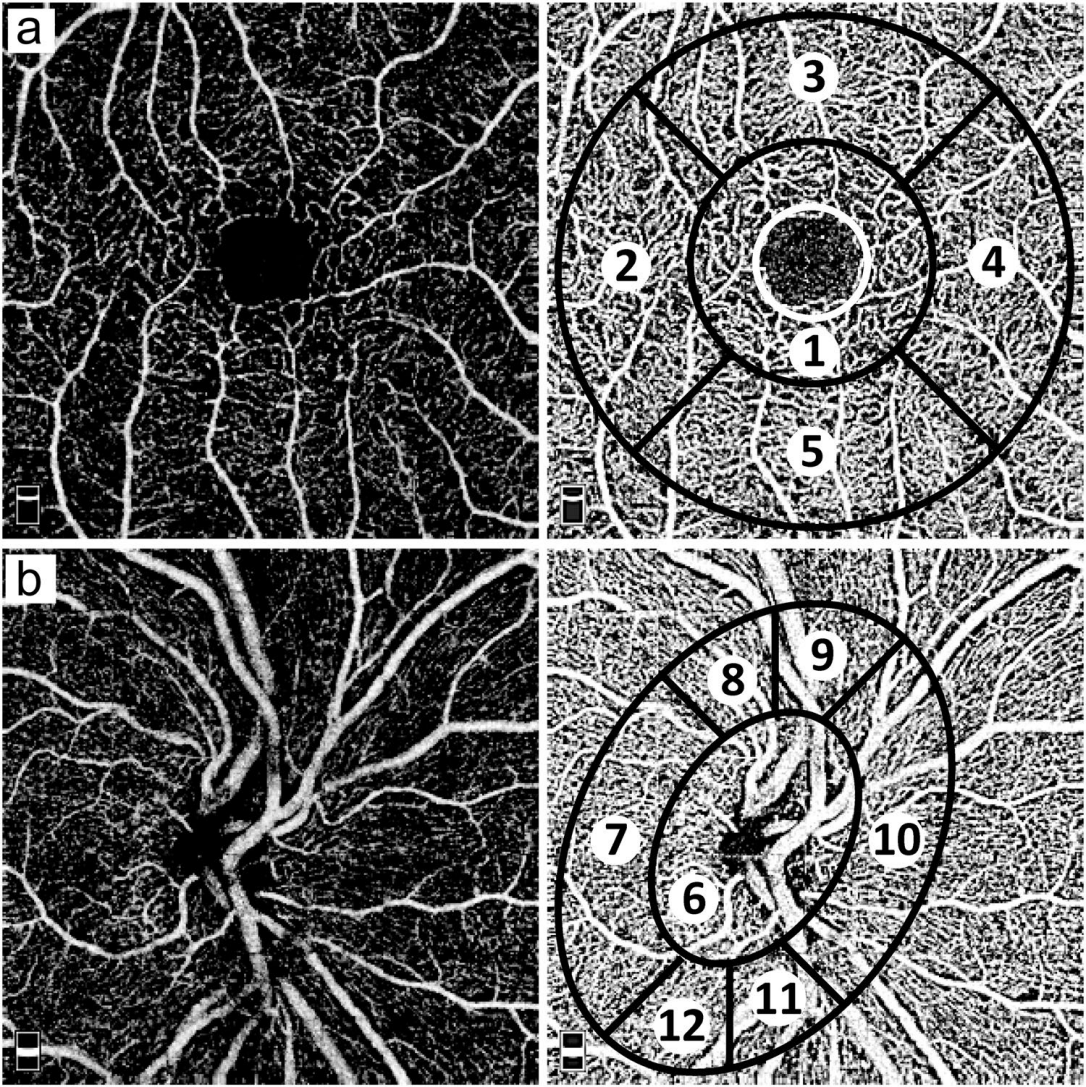
Original and schematic OCTA images. (**a**) superficial retina angiogram containing: 1 = fovea (diameter 1 mm, small white circle: foveal avascular zone), 2 = temporal, 3 = superior, 4 = nasal, 5 = inferior; parafovea = sum of 2–5, diameter 2.5 mm), (**b**) optic nerve head (6 = inside disc, 7 = nasal, 8 = superior nasal, 9 = superior temporal, 10 = temporal, 11 = inferior temporal, 12 = inferior nasal). “Whole en face” is defined by the average VD of all sections.

General eye health was routinely assessed and no differences were observed between the groups regarding eye diseases and subjective afflictions concerning the eye before start of measurements. Ocular examinations including visual field testing (automated Humphrey Visual Field Analyzer II, model 750, Carl Zeiss Meditec AG, Jena, Germany) and confocal scanning laser tomography of the optic disc was also performed (HRT III, Heidelberg Retina Tomograph, Heidelberg Engineering, Heidelberg, Germany). In all cases, an experienced examiner manually marked the optic disc margin on the mean topographic image, and the software then automatically calculated the optic disc measurements. Imaging of the cornea was performed by a Scheimpflug tomography (Pentacam HR, Oculus, Wetzlar, Germany). Rotating around the eye, the slit-camera device generates a series of radially oriented images to measure the anterior and posterior corneal surfaces. Based on obtained images, a 3D model of the corneal structure is generated. In the subsequent analysis of the sectional images, tissue boundaries are detected and point clouds are assigned to the various tissue layers. Only Pentacam images of good quality (internal quality indicator of the Pentacam) were included. OCTA^34,35^ using the RTVue XR Avanti with AngioVue (Optovue Inc., Fremont, California, USA) was used to image three times the macula using a 3 × 3 mm² scan (Fig. 4A) and the optic nerve head using a 4.5 × 4.5 mm² scan (Fig. 4B). Discrimination into foveal and parafoveal areas is performed by the AngioVue software that automatically provides and places the template into the centre of the fovea. The AngioVue software uses the ETDRS grid configuration. The diameter of the inner circle (corresponding to the foveal area) is 1 mm, and the thickness of the ring designating parafoveal area is 0.75 mm, leading to a total diameter of the circle surrounding parafoveal area of 2.5 mm. Accuracy of automatic segmentation of the retinal tissue in the macula into superficial vascular layer, deep vascular layer, outer retina, and CC was checked before analysis. Vessel density (VD) values for the superficial and deep retinal layers were used as provided by the automated AngioVue software. For VD values of the CC, images were exported and further analysed using Adobe Photoshop CS6, Adobe Systems, Inc., California, USA, by converting them into grey scales and attributing each pixel to a value that represents the strength of the decorrelation signal. VD was defined as the average decorrelation value of all pixels in the CC images.

Only images with good quality, without motion artefacts and a signal strength index of ≥60 were included. Mean values from three measurements were used for further analyses. The size of the fovea avascular zone (FAZ, white circle in Fig. 4A) was assessed using the OCT software.

### Normalization of Retinal Vessel Density

Values of VD in the region of the fovea given by the AngioVue device (“VD_given_”) are technically limited because there are no vessels in the FAZ, thus pretending a lower value than if the same level of vascularisation would be present in the whole area of the image. Therefore, in order to get the “real” values of VD (“VD_real_”) of the vascularized part of the imaged field of the fovea without the FAZ, we converted the given VD values by the following formula:

1 $$V{D}_{real}=\frac{V{D}_{given}\ast are{a}_{image}}{are{a}_{image}-are{a}_{FAZ}}$$

For easy reading, VD_real_ values of the fovea given in this paper are called only VD values. Segmentation analysis was used to determine thickness of single retinal layers. Automatic segmentation provided by the AngioVue software was checked, and thickness of the single layers was measured manually by an experienced examiner using the built-in measuring tool.

### Statistical Analyses

Microsoft Excel 2010 was used for data management. Statistical analyses were performed using GraphPad Prism 6.0 (GraphPad Software, Inc., La Jolla, California, USA). Data of KS patients were compared with male and female control cohorts using Mann–Whitney *U* test. Values are given as median and interquartile range (IQR). The general significance level was set to 0.05 (*p < 0.05, **p < 0.01, ***p < 0.001).

### Data availability statement

The corresponding author had full access to all the data in the study and all authors shared final responsibility for the decision to submit for publication.

## Electronic supplementary material

Supplementary Information
