# Supplementary material for: Aberrant ocular architecture and function in patients with Klinefelter syndrome
Source: Sci Rep. 2017 Oct 13;7:13130. doi: 10.1038/s41598-017-13528-4 (PMC5640645; doi:10.1038/s41598-017-13528-4)
Supplement: Supplementary file 1 — Supplementary Information [file 41598_2017_13528_MOESM1_ESM.pdf]

Supplement

Aberrant ocular architecture and function in patients with Klinefelter syndrome

Cristin Brand, MSc[1]; Michael Zitzmann, MD[2]; Nicole Eter, MD[3]; Sabine Kliesch, MD[2]; Joachim Wistuba, PhD[1]; Maged Alnawaiseh, MD[3†]; Peter Heiduschka, PhD[3†]*

Supplementary Table S1

		women (n=38)	KS patients (n=21)	men (n=26)
superficial	whole en face	54 (52-55)	53 (52-54)	53 (49-55)
	fovea[1]	47 (44-49)	47 (44-48)	46 (44-50)
	parafovea	56 (53-57)	55 (54-56)	54 (52-57)
	temporal	55 (52-55)	53 (51-54)	54 (51-56)
	superior	56 (54-58)	55 (53-57)	54 (52-58)
	nasal	55 (53-56)	54 (53-55)	54 (52-56)
	inferior	56 (55-58)	56 (53-57)	55 (53-58)
deep	whole en face	58 (57-60)*	57 (57-59)	57 (56-60)
	fovea[1]	55 (50-60)	52 (50-60)	55 (52-58)
	parafovea	61 (60-62)**	59 (57-61)	60 (58-61)[+]
	temporal	60 (59-61)**	58 (56-60)	59 (57-61)
	superior	62 (61-63)**	60 (58-62)	61 (58-63)
	nasal	60 (59-62)	59 (57-61)	60 (58-61)
	inferior	61 (61-63)**	60 (59-62)	61 (60-63)

Supplementary Table S2

	women (n=22)	KS patients (n=19)	men (n=11)
disc area [mm^2]	1.75 (1.47-1.98) *	1.93 (1.72-2.43)	1.77 (1.49-2.12)
cup area [mm^2]	0.37 (0.21-0.57)	0.30 (0.13-0.63)	0.29 (0.07-0.61)
rim area [mm^2]	1.39 (1.19-1.52) **	1.60 (1.35-1.76)	1.43 (1.24-1.61)
mean cup depth [mm]	0.19 (0.12-0.23)	0.16 (0.11-0.18)	0.16 (0.09-0.22)
max cup depth [mm]	0.53 (0.42-0.65)	0.44 (0.32-0.58)	0.44 (0.21-0.60)
mean RNFL thickness [mm]	0.25 (0.22-0.29)	0.25 (0.20-0.30)	0.23 (0.17-0.27)

Supplement legends

Figure S1: Features of the optical nerve head (ONH) in KS patients versus controls. (**a**) Vessel densities (VD) at ONH, (**b**) disc area and (**c**) rim area in KS patients and controls. Levels of significance of differences between controls and KS patients are indicated by p<0.05=* and p<0.01=**. "+" indicates statistically significant sex-related differences between control cohorts.

Table S1: Overview of VD values sorted by layers and regions. "Whole en face" defines the overall VD of the angiogram, which is further divided into "fovea" and "parafovea". [1]Foveal values are normalized for FAZ areas. Values for "parafovea" were further divided into four subsectors (temporal, superior, nasal and inferior). Levels of significance of differences between controls and KS patients are indicated by p<0.05=* and p<0.01=**. "+" indicates statistically significant sex-related differences between control cohorts.

Table S2: Parameters of the optic nerve head analysed in KS patients and controls (RNFL: retinal nerve fiber layer). Levels of significance of differences between controls and KS patients are indicated by p<0.05=* and p<0.001=**.

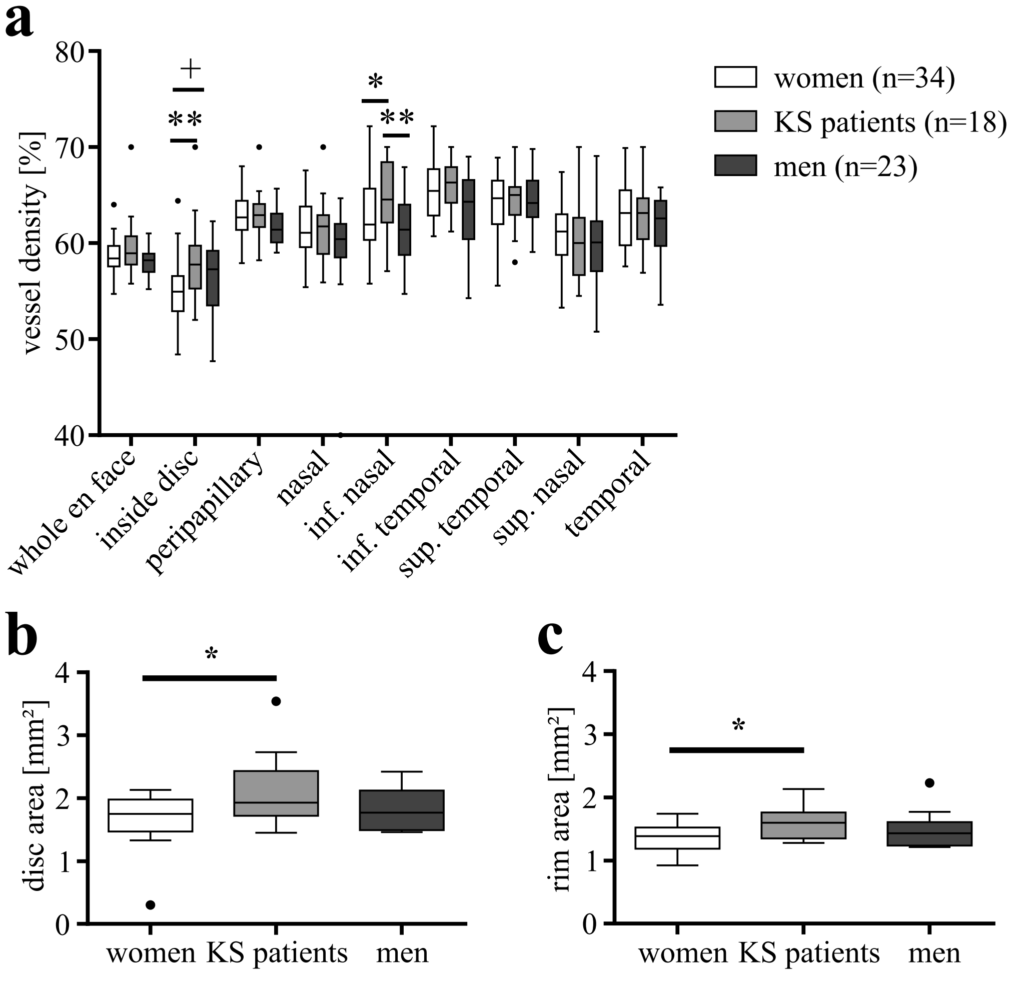
a
vessel density [%]
80
70
60
50
40
+
* *
*
* *
women (n=34)
KS patients (n=18)
men (n=23)
whole en face
inside disc
peripapillary
nasal
inf. nasal
inf. temporal
sup. temporal
sup. nasal
temporal
b
disc area [mm²]
4
3
2
1
0
*
women
KS patients
men
c
rim area [mm²]
4
3
2
1
0
*
women
KS patients
men
